# Long noncoding RNA LINC01391 restrained gastric cancer aerobic glycolysis and tumorigenesis via targeting miR-12116/CMTM2 axis

**DOI:** 10.7150/jca.48365

**Published:** 2020-08-27

**Authors:** Cuijuan Qian, Zhurong Xu, Luyan Chen, Yichao Wang, Jun Yao

**Affiliations:** 1 Institute of Tumor, School of Medicine, Taizhou University, Taizhou, Zhejiang 318000, China.; 2 Department of Medical Laboratory, Taizhou Central Hospital, Taizhou University Hospital, Taizhou, Zhejiang 318000, China.

**Keywords:** gastric cancer, glycolysis, LINC01391, CMTM2, miR-12116

## Abstract

Increasing studies indicate that long noncoding RNA (lncRNA) plays a critical role in aerobic glycolysis of various tumors. However, the contribution of lncRNA in gastric cancer (GC) cell glycolysis is still poorly understood. The objective of this research was to investigate the functional role and mechanism of lncRNA long intergenic non-protein coding RNA 1391 (LINC01391) in the aerobic glycolysis and tumorigenesis of GC. Here, we report that LINC01391 was low expressed in GC tissues and cell lines. LINC01391 overexpression hampered GC cell proliferation, migration, invasion and aerobic glycolysis, while LINC01391 knockdown demonstrated the opposite effects. LINC01391 overexpression delayed the tumor growth *in vivo*. Furthermore, LINC01391 interacted with miR-12116, and miR-12116 interacted with CMTM2 in GC cells. And miR-12116 and CMTM2 participated in the inhibitory effects of LINC01391 on cell migration, invasion and aerobic glycolysis in GC cells. LINC01391 restrained aerobic glycolysis and tumorigenesis of GC via targeting miR-12116/CMTM2 axis, which provides new insights into mechanism of GC progression.

## Introduction

Gastric cancer (GC) is a digestive system malignant tumor with the highest morbidity and mortality rates in the world 1,2. In spite of the development of surgery, chemotherapy and radiotherapy, the overall survival (OS) of GC patients remains unimproved over the last decades because the molecular mechanisms of GC occurrence and progression are poorly understood 1-3. Thus, it is great significant to explore the molecular mechanisms of GC occurrence and development, and identify novel diagnostic and prognostic biomarkers and therapeutic targets for GC.

Recently, an increasing number of studies demonstrated that long noncoding RNAs (lncRNAs) may exert critical roles in GC occurrence and development 4,5 and microRNAs (miRNAs) function as essential regulators via involvement in various signaling transduction pathways in GC progression 6. Moreover, increasing studies have demonstrated that a number of lncRNAs function as competitive endogenous RNAs (ceRNA), by which lncRNAs can restore the repression of downstream target genes via sponging miRNAs in GC cells 4,7. Interestingly, lncRNA long intergenic non-protein coding RNA 1391 (LINC01391; NCBI Accession no. 103344930) has been reported to exert an anti-oncogenic role via attenuating cell proliferation and invasion in hepatocellular carcinoma (HCC) 8. However, up to data, the role of LINC01391 has not been systematically studied in GC. Therefore, we here provided strong evidences to support the function and related mechanism of LINC01391 in GC progression.

Here, we aimed to investigate the functional involvement of a novel anti-oncogenic lncRNA, LINC01391, in cell invasion, migration and aerobic glycolysis in GC. Furthermore, we focused on the interaction between LINC01391 and miR-12116, as well as that between miR-12116 and CMTM2 (CKLF like MARVEL transmembrane domain containing 2), a potential target gene of miR-12116. Our findings in this study may be helpful for enlarging our insight into the tumor metabolism and pathogenesis of GC.

## Materials and Methods

### Patients and samples

The present research was conducted with the approval of the Human Ethics Committee of Taizhou University Hospital, and each patient signed the written informed consent before surgery. The forty paired GC tissues samples and their paracancerous nontumor samples were all collected from Taizhou University Hospital (Taizhou, Zhejiang, China). The criteria for case selection were as follows: All specimens were diagnosed for primary gastric carcinoma by more than two pathologists. All the patients enrolled were adults (older than 18 years old) with no history of other cancer, and had not undergone any targeted therapeutic treatments prior to sample collection. Patient characteristics and clinical findings are detailed in Table 1. All fresh samples were immediately snap-frozen in liquid nitrogen and preserved at -80 °C before use.

### Cell culture and transfection

The human normal gastric epithelial cell line GES-1 and human GC cell lines (SGC7901, AGS, MKN45 and BGC823) were all obtained from American Type Culture Collection (ATCC, Manassas, VA, USA). All cell lines used in this study were cultured in RPMI 1640 (Gibco, NY, USA) supplemented with 10% FBS (Gibco, NY, USA) in a humidified 5% CO_2_ atmosphere at 37 °C. The GC cells were transfected with 100 ng of the vector or 50 nM of the indicated oligonucleotide by using Lipofectamine 3000 reagent (Invitrogen, CA, USA) as per the standard test protocols.

### Quantitative real-time PCR (qRT-PCR)

Total RNA of GC cells and tissues was extracted using the TRIzol reagent (Invitrogen, CA, USA), respectively. The first-strand cDNA was synthesized using the reverse transcriptase cDNA synthesis kit (Takara, Dalian, China). qRT-PCR was performed with the SYBR Green qPCR Kit (Thermo Fisher Scientific, Waltham, MA, USA) using a StepOne™ Real-Time PCR System (Applied Biosystems, Carlsbad, CA, USA). The relative quantification of genes was carried out via the 2^-ΔΔCt^ method and normalized to the internal control GAPDH or U6. Sequences of all primers used in the experiment are listed in Table 2, and the RNA expression levels were analyzed as described previously 8.

### Cell proliferation assay

After transfection, GC cell viability was assessed with the Cell Counting Kit-8 (CCK-8) kit (Abcam, Cambridge, MA, USA). In brief, cells (3 × 10^3^ cells/200 μl/well) were seeded into the 96-well plates, and six replicates were set up for each sample. 100 μl of fresh medium were added to replace culture medium at the appointed time point, and 10 μl of CCK-8 solution were pipetted into each well immediately. After incubated at 37 °C for 2 h, the OD450 value was determined using a microplate reader (Bio-Rad Laboratories, Inc., Hercules, CA, USA).

### Cell migration and invasion assay

For cell migration assay, GC cells (5 × 10^4^ cells/250 μl/well) were resuspended in RPMI 1640 containing 1% FBS and seeded into uncoated 8-μm transwell filter inserts (Corning, USA) in triplicate. 600 μL of RPMI 1640 supplemented with 15% FBS were pipetted into the lower chambers as a chemoattractant. After incubation for 16 hours, the non-migratory GC cells in the upper chamber were removed via a cotton swab, and the migrated cells on the bottom of the membrane were fixed with 100% methanol. Fixed cells were stained with 0.5 μg/ml 4′,6-diamidino-2-phenylindole at room temperature for 5 min, and then counted under a fluorescence microscope (Eclipse 80i; Nikon Corporation, Tokyo, Japan) in five random fields.

For cell invasion assay, the upper chambers of transwell inserts were coated with Matrigel (1:8, BD Biosciences, Franklin Lakes, NJ, USA). GC cells were allowed to invade the chambers coated with matrigel for 48 h. The invaded cells on the bottom of the membrane were stained using 0.1% crystal violet and then the membrane was incubated in 200 μl of lysis reagent at room temperature. Finally, 100 μl of lysate was transferred to a 96-well plate for OD450 value determine via a microplate reader (550; Bio-Rad, USA).

### Glucose uptake and lactate production assay

After transfection, GC cells were cultured in glucose-free culture medium for 16 h, and then replaced by high-glucose culture medium under normoxic conditions for an additional 24 h. The supernatants of GC cell culture medium and the GC cells were collected separately. The glucose uptake and lactate production levels were measured via a glucose assay kit (BioVision, Milpitas, California, USA) and a lactate assay kit (BioVision) according to the manufacturer's instructions, respectively.

### Western blotting

After transfection, proteins from cultured GC cells were extracted using RIPA buffer (Beyotime, Shanghai, China), and the protein concentration was measured via the BCA Kit (Pierce, Rockford, IL, USA). Then the proteins were resolved using the SDS-PAGE Electrophoresis System and wet transferred to PVDF membranes (Millipore, Bedford, MA, USA). Next, the membranes were blocked at room temperature in 5% non-fat milk, followed by incubation with specific primary antibodies at 4 °C overnight. Primary antibodies against CMTM2 (1:1000, #NBP1-59439, Novus), LDH-A (1:1000, #ab125683, Abcam), GLUT1 (1:1000, #ab15309, Abcam), and GAPDH (1:1000, #ab181602, Abcam) were used. And the second day, the membranes were washed and then incubated with horseradish peroxidase-conjugated rabbit secondary antibody (1:2000, #7074, Cell Signaling Technology) at room temperature for 1 hour. The interest protein bands were visualized using an enhanced chemiluminescence system (ECL kit; Pierce Biotechnology Inc., Rockford, IL, USA) and then scanned via an LAS-4000 imaging system (Fujifilm Holdings Corporation, Tokyo, Japan).

### Pull-down assay with biotinylated miR-12116

48 hours after transfection with biotinylated wild type (wt) miR-12116 (Bio-miR-12116-WT), mutated (mt) miR-12116 (Bio-miR-12116-MT) or antagonistic miR-12116 probe (GenePharma, Shanghai, China), SGC7901 cells were collected and lysed in specific lysis buffer (Ambion, Austin, TX, USA) for 10 min, and then mixed with M-280 streptavidin magnetic beads (Sigma-Aldrich, St. Louis, MO, USA) for 3 hours at 4 °C. TRIzol reagent (Invitrogen, CA, USA) was used to elute and purify the interacted RNA complex, and qRT-PCR was used to detect the expression level of LINC01391.

### Luciferase reporter assay

To generate wild-type LINC01391 reporter (LINC01391-WT) or CMTM2 reporter (CMTM2-WT), the partial sequences of LINC01391 or CMTM2 3′-untranslated region (UTR), which contains the putative miR-12116-binding site, were amplified via PCR and constructed into the pmirGLO Luciferase vector (Promega, Madison, WI, USA). The mutant-type LINC01391 (miR-12116 target site-mutation LINC01391, LINC01391-MUT) reporter or mutant-type CMTM2 (miR-12116 target site-mutation CMTM2 3′-UTR, CMTM2-MUT) reporter were produced using GeneArt™ Site-Directed Mutagenesis System (Thermo Fisher Scientific, Waltham, MA, USA). All constructs were verified via DNA sequencing. Subsequently, the luciferase reporter and miR-12116 mimic or control mimic were co-transfected into SGC7901 cells. After 48 hours of transfection, luciferase activity was evaluated via a dual-luciferase reporter assay system (Promega, Madison, WI, USA) according to the manufacturer's instructions.

### Animal experiments

Animal experiments were approved by the Committee on Ethics of Animal Experiments of Taizhou University Medical School. Female athymic nude mice (4-week-old) were obtained from Shanghai Laboratory Animal Center (Chinese Academy of Sciences, China), and randomized and assigned to different groups (5 per group). SGC7901 cells transfected with si-NC or si-LINC01391 and non-transfected SGC7901 cells (blank control) were collected and resuspended at a concentration of 2 × 10^6^ cells/200 μl. The cells were injected subcutaneously into the right side of the back of the mice, separately. The diameter of each xenograft tumor was measured using a vernier caliper every 3 days. The tumour volume was calculated regularly according to the formula: Volume of tumor (mm^3^) = (length × width^2^)/2. The animals were finally euthanised on day 24 after injection.

### Statistical analyses

Data are expressed as mean ± standard deviation (SD). SPSS 18.0 software (SPSS Inc., IL, USA) was used to perform statistical analysis. The associations between LINC01391, miR-12116 and CMTM2 expression were analyzed using Pearson correlation. Survival analysis was conducted using the Kaplan-Meier method and analyzed via the log-rank test. Statistical differences were calculated with one-way ANOVA analysis. *P*-values less than 0.05 were considered statistically significant.

## Results

### LINC01391 expression is decreased in GC tissues and cell lines

**Figure 1:**
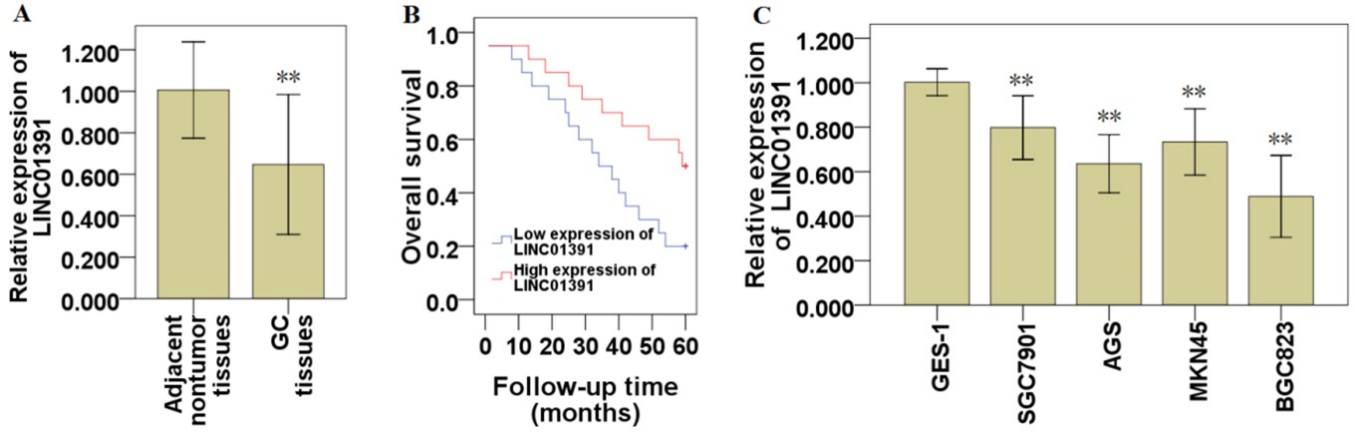
** LINC01391 expression in GC tissues and cell lines.** The expression of LINC01391 in GC and adjacent nontumor tissues was identified via qRT-PCR (**A**). Overall survival rates of GC patients with high and low LINC01391 expression were analyzed via Kaplan-Meier survival analysis (**B**). The expression of LINC01391 in GC cell lines and GES-1 cells was detected via qRT-PCR (**C**). **P < 0.01 vs control.

To explore the expression of LINC01391 in GC, we detected the expression levels of LINC01391 in GC tissues and cell lines via qRT-PCR. The current results demonstrated that LINC01391 expression was significantly decreased in GC tissues compared with that in paired adjacent nontumor samples (Figure 1A). The clinicopathological characteristics of the 40 GC patients were shown in Table 1. Low expression of LINC01391 was found to be significantly associated with lymph node metastasis, advanced TNM stage and tumor size. Furthermore, low expression of LINC01391 predicted an unfavourable prognosis (Figure 1B). On the other hand, LINC01391 expression was significantly decreased in GC cell lines, as compared with GES-1 (Figure 1C). The above findings suggest a potential role of LINC01391 as a tumor-suppressive lncRNA in GC progression.

### LINC01391 modulates the cell proliferation of GC cells

**Figure 2:**
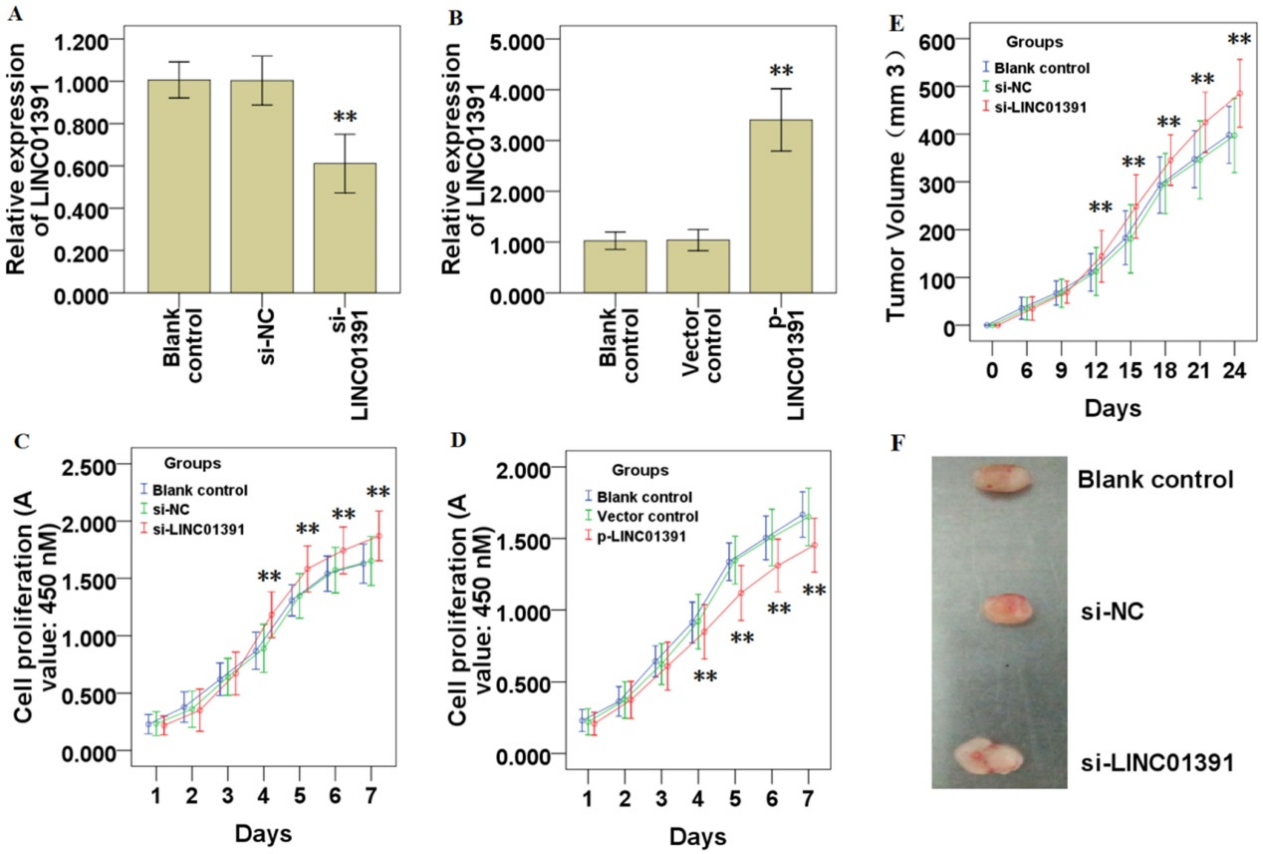
** LINC01391 modulates GC cell proliferation *in vitro* and *in vivo*.** LINC01391 siRNA (si-LINC01391) transfection silenced the LINC01391 expression in SGC7901 cells (**A**). pcDNA3.1-LINC01391 (p-LINC01391) transfection enforced the LINC01391 expression in BGC823 (**B**). CCK-8 assay was performed to detect the cell proliferative ability of SGC7901 cells with LINC01391 knockdown (**C**). CCK-8 assay was performed to detect the cell proliferative ability of BGC823 cells with LINC01391 overexpression (**D**). *In vivo* heterotransplantation mice assay illustrated the tumor volume of SGC7901 cell neoplasm (**E**). Representative figure of tumors harvesting from the nude mice was shown (**F**). **P < 0.01 vs control.

The gain and loss of functional experiments were used to determine the biological functions of LINC01391. qRT-PCR analysis demonstrated LINC01391 was significantly knocked down via si-LINC01391 in SGC7901 cells (Figure 2A), and overexpressed via p-LINC01391 in BGC823 cells (Figure 2B). CCK-8 assay demonstrated that LINC01391 knockdown significantly accelerated the cell proliferation of SGC7901 cells (Figure 2C), while LINC01391 overexpression significantly repressed the cell proliferation of BGC823 cells (Figure 2D). Furthermore, *in vivo* heterotransplantation mice assay illustrated that LINC01391 knockdown significantly elevated the tumor volume of SGC7901 cells (Figure 2E and 2F). Overall, the above findings indicated that LINC01391 negatively modulated the cell proliferation of GC cells *in vitro* and *in vivo*.

### LINC01391 modulates the cell invasion and migration of GC cells

**Figure 3:**
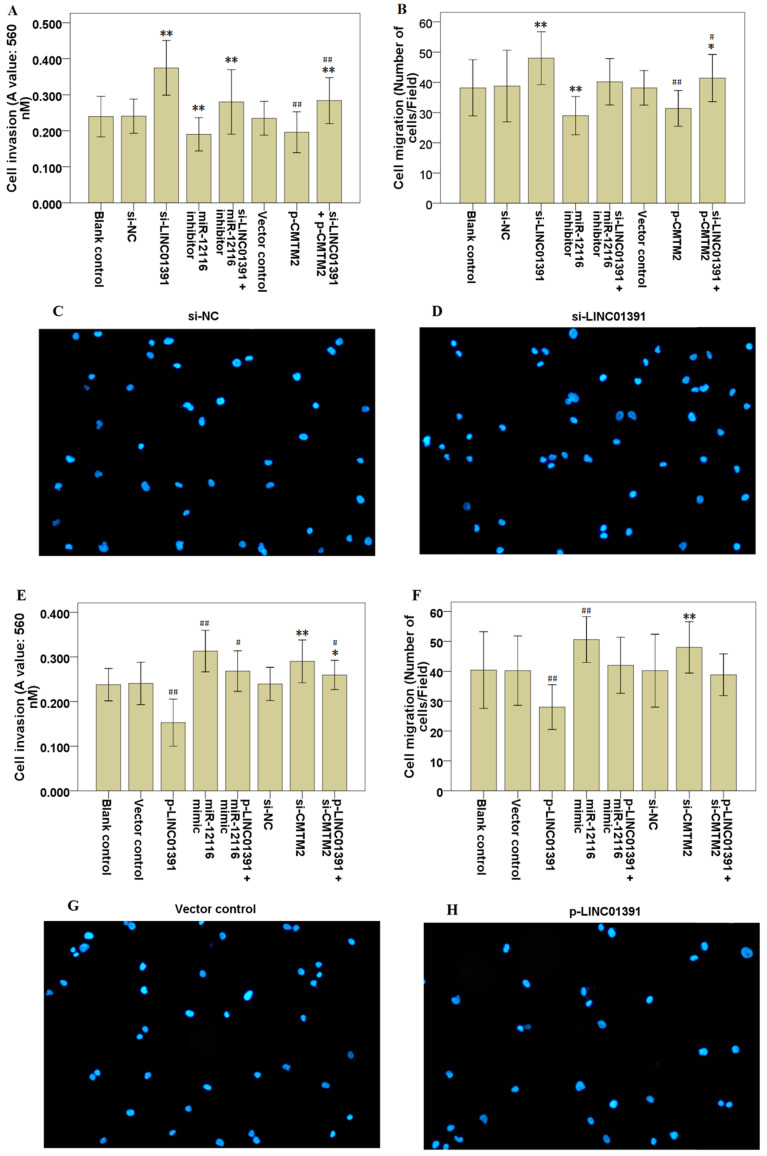
** LINC01391 modulates GC cell invasion and migration via miR-12116 and CMTM2.** After transfections with si-NC, si-LINC01391, miR-12116 inhibitor, si-LINC01391 + miR-12116 inhibitor, vector control, p-CMTM2, or si-LINC01391 + p-CMTM2, transwell assay with Matrigel was performed to detect the cell invasion ability following the transfections in SGC7901 cells (**A**), and transwell assay without Matrigel was performed to detect the cell migration ability following the transfections in SGC7901 cells (**B**). Representative pictures of the cell migration in SGC7901 cells were manifested (**C,D**). After transfections with vector control, p-LINC01391, miR-12116 mimic, p-LINC01391 + miR-12116 mimic, si-NC, si-CMTM2, or p-LINC01391 + si-CMTM2, transwell assay with Matrigel was performed to detect the cell invasion ability following the transfections in BGC823 cells (**E**), and transwell assay without Matrigel was performed to detect the cell migration ability following the transfections in BGC823 cells (**F**). Representative pictures of the cell migration in BGC823 cells were manifested (**G,H**). *P < 0.05, **P < 0.01 vs si-NC; ^#^*P* < 0.05, ^##^*P* < 0.01 vs vector control.

Moreover, transwell assay with or without Matrigel demonstrated that LINC01391 knockdown significantly accelerated the cell invasion (Figure 3A) and migration (Figure 3B-D) of SGC7901 cells, while LINC01391 overexpression significantly repressed the cell invasion (Figure 3E) and migration (Figure 3F-3H) of BGC823 cells. Overall, these findings indicated that LINC01391 negatively modulated the cell invasion and migration of GC cells.

### LINC01391 modulates the glucose uptake and lactate production of GC cells

**Figure 4:**
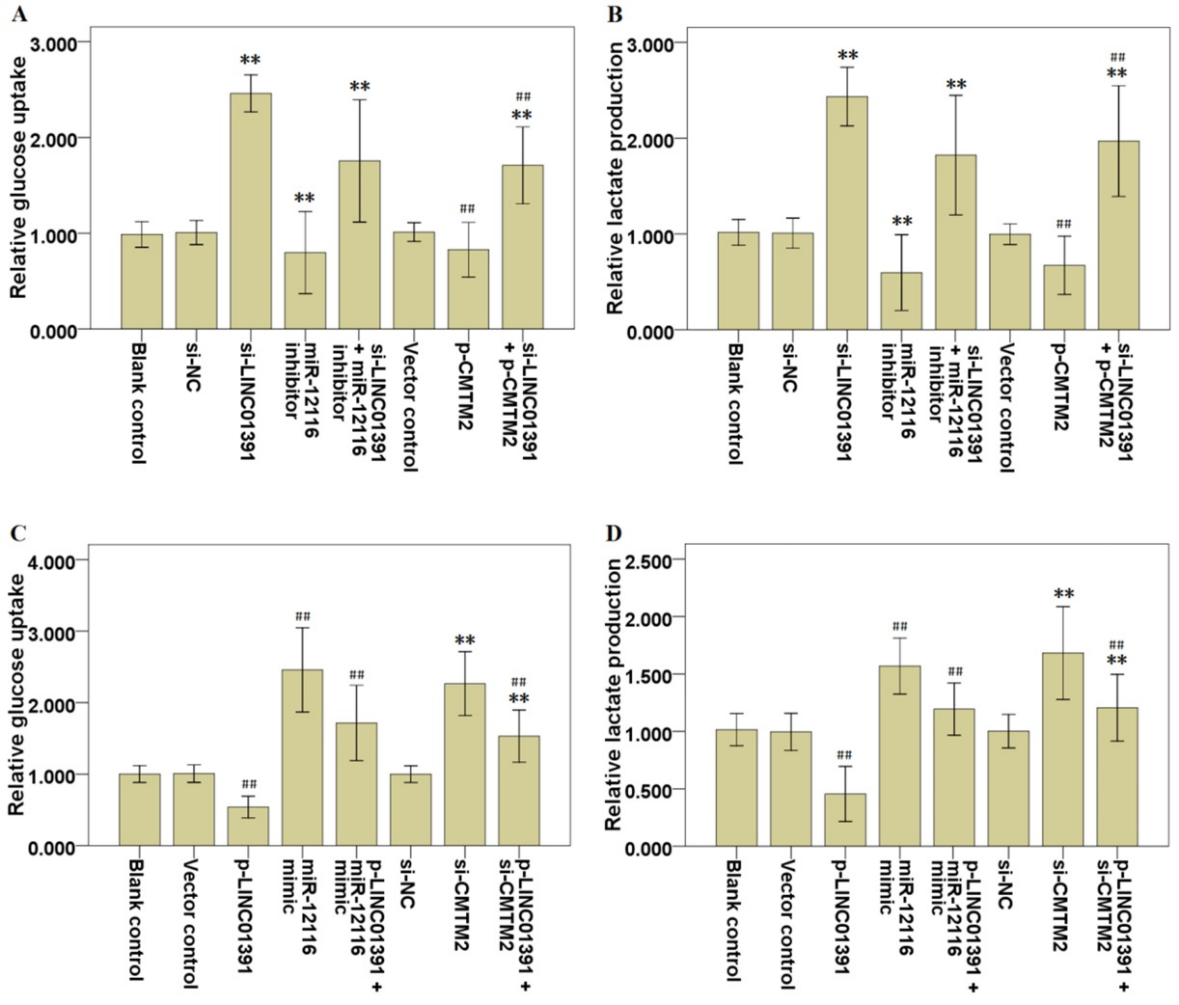
** LINC01391 modulates the glucose uptake and lactate production of GC cells via miR-12116 and CMTM2.** After transfections with si-NC, si-LINC01391, miR-12116 inhibitor, si-LINC01391 + miR-12116 inhibitor, vector control, p-CMTM2, or si-LINC01391 + p-CMTM2, glucose uptake levels were detected via glycolysis analysis following the transfections in SGC7901 cells (**A**), and lactate production levels were detected via glycolysis analysis following the transfections in SGC7901 cells (**B**). After transfections with vector control, p-LINC01391, miR-12116 mimic, p-LINC01391 + miR-12116 mimic, si-NC, si-CMTM2, or p-LINC01391 + si-CMTM2, glucose uptake levels were detected via glycolysis analysis following the transfections in BGC823 cells (**C**), and lactate production levels were detected via glycolysis analysis following the transfections in BGC823 cells (**D**). **P < 0.01 vs si-NC; ^##^*P* < 0.01 vs vector control.

The glycolysis analysis was applied to identify the effects of LINC01391 expression on glucose uptake and lactate production, and the results demonstrated that LINC01391 knockdown significantly elevated the glucose uptake (Figure 4A) and lactate production (Figure 4B) in SGC7901 cells, while LINC01391 overexpression significantly restrained the glucose uptake (Figure 4C) and lactate production (Figure 4D) in BGC823 cells. Overall, these findings indicated that LINC01391 negatively modulated the glucose uptake and lactate production of GC cells.

### LINC01391 modulates the GLUT1 and LDH-A expression in GC cells

**Figure 5:**
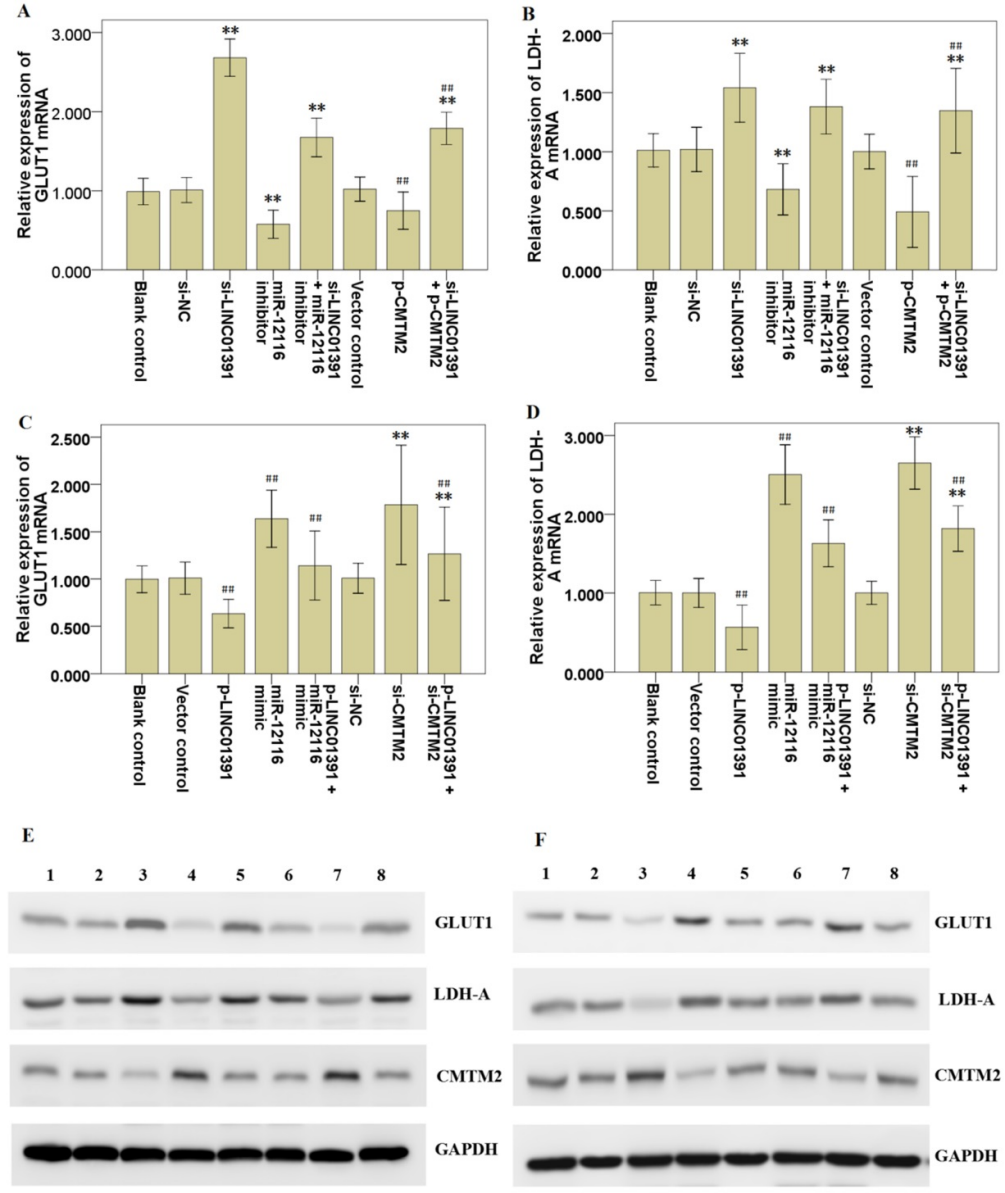
** LINC01391 modulates the GLUT1 and LDH-A expression in GC cells via miR-12116 and CMTM2.** After transfections with si-NC, si-LINC01391, miR-12116 inhibitor, si-LINC01391 + miR-12116 inhibitor, vector control, p-CMTM2, or si-LINC01391 + p-CMTM2, GLUT1 mRNA expression levels were detected via qRT-PCR analysis following the transfections in SGC7901 cells (**A**), and LDH-A mRNA expression levels were detected via qRT-PCR analysis following the transfections in SGC7901 cells (**B**). After transfections with vector control, p-LINC01391, miR-12116 mimic, p-LINC01391 + miR-12116 mimic, si-NC, si-CMTM2, or p-LINC01391 + si-CMTM2, GLUT1 mRNA expression levels were detected via qRT-PCR analysis following the transfections in BGC823 cells (**C**), and LDH-A mRNA expression levels were detected via qRT-PCR analysis following the transfections in BGC823 cells (**D**). Western blot analysis of GLUT1, LDH-A and CMTM2 expression following the transfections in SGC7901 cells (**E**). Lanes 1, Blank control; Lanes 2, si-NC; Lanes 3, si-LINC01391; Lanes 4, miR-12116 inhibitor; Lanes 5, si-LINC01391 + miR-12116 inhibitor; Lanes 6, Vector control; Lanes 7, p-CMTM2; Lanes 8, si-LINC01391 + p-CMTM2. Western blot analysis of GLUT1, LDH-A and CMTM2 expression following the transfections in BGC823 cells (**F**). Lanes 1, Blank control; Lanes 2, Vector control; Lanes 3, p-LINC01391; Lanes 4, miR-12116 mimic; Lanes 5, p-LINC01391 + miR-12116 mimic; Lanes 6, si-NC; Lanes 7, si-CMTM2; Lanes 8, p-LINC01391 + si-CMTM2. GAPDH was used as an internal control to show equal protein loading. **P < 0.01 vs si-NC; ^##^*P* < 0.01 vs vector control.

qRT-PCR and Western blotting analysis were performed to detect the mRNA and protein expression levels of aerobic glycolysis associated proteins, including GLUT1 and LDH-A. Our present results revealed that LINC01391 knockdown significantly elevated GLUT1 (Figure 5A) and LDH-A (Figure 5B) mRNA expression in SGC7901 cells. In contrast, LINC01391 overexpression significantly decreased GLUT1 (Figure 5C) and LDH-A (Figure 5D) mRNA expression in BGC823 cells. Consistently, Western blotting analysis demonstrated that LINC01391 knockdown obviously elevated protein expression of GLUT1 and LDH-A in SGC7901 cells (Figure 5E), whereas LINC01391 overexpression yielded the opposite effects in BGC823 cells (Figure 5F). These above results suggested that LINC01391 might regulate the aerobic glycolysis via modulating GLUT1 and LDH-A expression in GC cells.

### Association of LINC01391, miR-12116 and CMTM2 in GC

**Figure 6:**
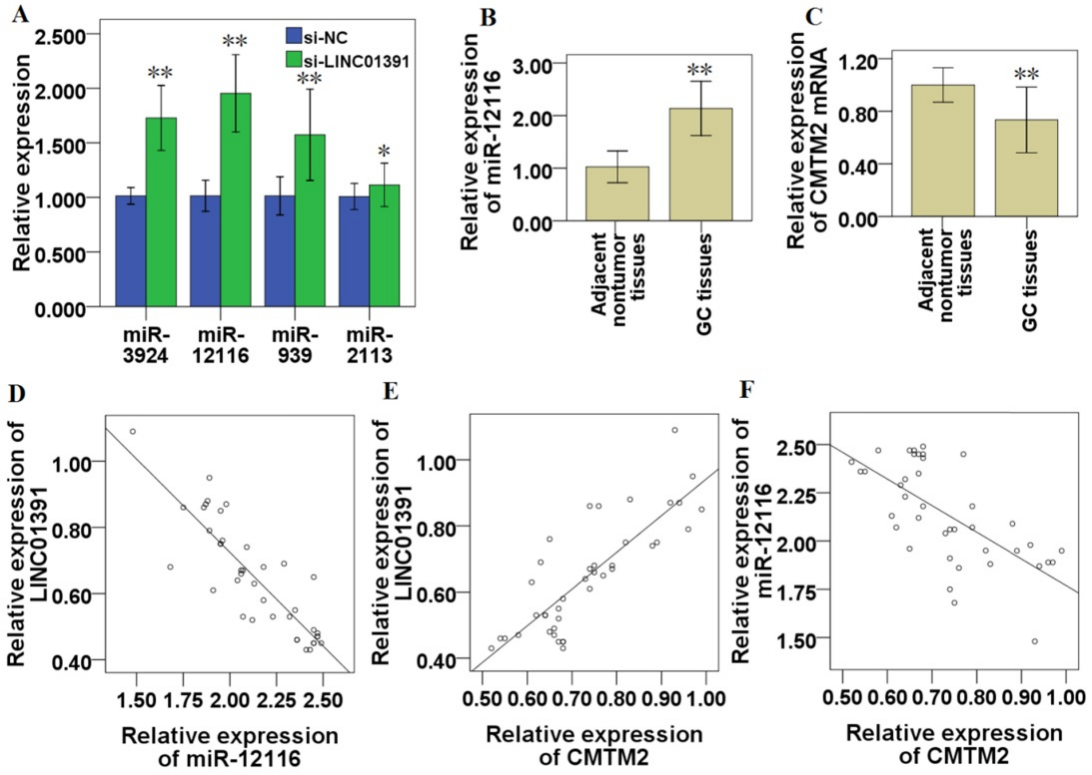
** Correlations between LINC01391, miR-12116 and CMTM2 in GC.** After transfection of si-LINC01391, the expressions of miR-3924, miR-12116, miR-939 and miR-2113 were detected via qRT-PCR (**A**). miR-12116 expressions in the GC and adjacent nontumor tissues were detected via qRT-PCR (**B**). CMTM2 expressions in the GC and adjacent nontumor tissues were detected via qRT-PCR (**C**). The correlation of the expressions between LINC01391 and miR-12116 was detected via correlation analysis (**D**). The correlation of the expressions between LINC01391 and CMTM2 mRNA was detected via correlation analysis (**E**). The correlation of the expressions between miR-12116 and CMTM2 was detected via correlation analysis (**F**). *P < 0.05, and **P < 0.01 vs control.

The potential target miRNAs of LINC01391 were predicted via two online bioinformatics database, miRDB (http://mirdb.org/) and TargetScanHuman (http://www.targetscan.org/vert_72/). The top four predicted potential target miRNAs of LINC01391 are miR-3924, miR-12116, miR-939 and miR-2113 (Table 3). The results of qRT-PCR showed that LINC01391 knockdown via si-LINC01391 accelerated the expressions of miR-3924, miR-12116, miR-939 and miR-2113, respectively, among which miR-12116 was the most obvious (Figure 6A). Further, potential target genes of miR-12116, that were likely to contribute to GC progression, were also predicted and listed in Table 4. The 3′-UTR of CMTM2 was predicted via bioinformatics analyses to have miR-12116-binding sites, so CMTM2 was chosen for our further studies.

miR-12116 and CMTM2 expression in the 40 specimens of GC tissues were detected via qRT-PCR, and the current results demonstrated that, compared with that in paired adjacent nontumor tissues, miR-12116 expression was significantly higher (n=40, *P* < 0.01; Figure 6B) and CMTM2 expression was significantly lower in GC tissues (n=40, *P* < 0.01; Figure 6C). After statistically analysis, LINC01391 was found to be inversely correlated with miR-12116 (*R*=-0.864, *P* < 0.01; Figure 6D), while positively correlated with CMTM2 expression (*R*=0.819, *P* < 0.01, Figure 6E). In addition, CMTM2 was inversely correlated with miR-12116 (*R*=-0.667, *P* < 0.01, Figure 6F).

### LINC01391 modulates the tumor phenotype of GC cells via miR-12116/CMTM2 axis

**Figure 7:**
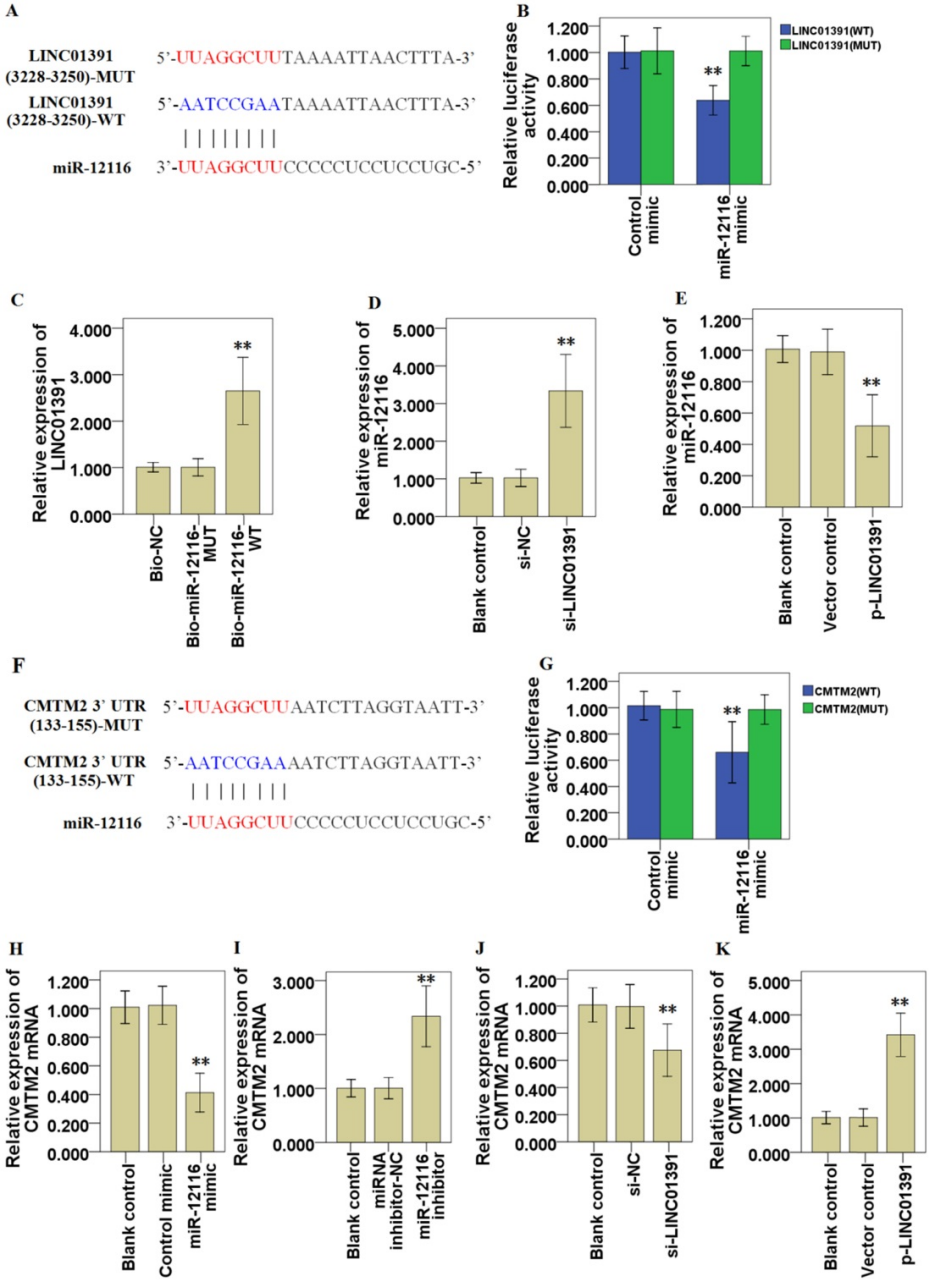
** LINC01391 modulates the tumor phenotype of GC cells via miR-12116/CMTM2 axis.** Bioinformatics analysis tools suggested that miR-12116 might share the binding sites with LINC01391 (**A**). Luciferase reporter assay indicated the molecular level combination of miR-12116 and LINC01391 (**B**). LINC01391 was pulled down via biotinylated miR-12116-WT (**C**). qRT-PCR determined the miR-12116 expression in SGC7901 cells transfected with si-NC or si-LINC01391 (**D**). qRT-PCR determined the miR-12116 expression in BGC823 cells transfected with empty vector (vector control) or p-LINC01391 (**E**). Bioinformatics analysis tools suggested the associability within miR-12116 and CMTM2 (**F**). Luciferase reporter assay indicated the covalent targeting of miR-12116 with CMTM2 mRNA 3′-UTR (**G**). qRT-PCR determined the CMTM2 mRNA expression in SGC7901 cells transfected with control mimic or miR-12116 mimic (**H**). qRT-PCR determined the CMTM2 mRNA expression in BGC823 cells transfected with miRNA inhibitor-NC (negative control) or miR-12116 inhibitor (**I**). qRT-PCR determined the CMTM2 mRNA expression in SGC7901 cells transfected with si-NC or si-LINC01391 (**J**). qRT-PCR determined the CMTM2 mRNA expression in BGC823 cells transfected with vector control or p-LINC01391 (**K**). **P < 0.01 vs control.

Online bioinformatics analysis via miRDB and TargetScanHuman suggested that miR-12116 might share the binding sites with LINC01391 (Figure 7A). Subsequently, the results from dual-luciferase reporter assay demonstrated that miR-12116 could bind with LINC01391 at the molecular level in SGC7901 cells (Figure 7B). To further validate the interaction between miR-12116 and LINC01391, RNA pull-down assay was performed using biotinylated miR-12116-WT and miR-12116-MUT probes. As shown in Figure 7C, only miR-12116-WT could bind and precipitate LINC01391 in cell lysates of SGC7901 cells. However, there was no interaction between LINC01391 and miR-12116-MUT or biotin-labeled antagonistic miR-12116 probe (Bio-NC). Moreover, qRT-PCR assay indicated that LINC01391 knockdown elevated miR-12116 expression in SGC7901 cells (Figure 7D), while LINC01391 overexpression repressed miR-12116 expression in BGC823 cells (Figure 7E). Therefore, we here found that LINC01391 had a direct effect on miR-12116.

Furthermore, the online bioinformatics analysis also suggested that miR-12116 had the higher associability with CMTM2, and miR-12116 might share the binding sites with CMTM2 3′-UTR (Figure 7F). Luciferase reporter assay confirmed that miR-12116 covalently targeted CMTM2 mRNA 3′-UTR in SGC7901 cells (Figure 7G). Thus, we here found that miR-12116 had a direct effect on CMTM2. Further, qRT-PCR assay indicated miR-12116 upmodulation repressed CMTM2 mRNA expression in SGC7901 cells (Figure 7H), while miR-12116 inhibition elevated CMTM2 mRNA expression in BGC823 cells (Figure 7I). And LINC01391 knockdown repressed CMTM2 mRNA expression in SGC7901 cells (Figure 7J), while LINC01391 overexpression elevated CMTM2 mRNA expression in BGC823 cells (Figure 7K). Western blot analysis demonstrated that LINC01391 knockdown repressed CMTM2 expression in SGC7901 cells (Figure 5E), while LINC01391 overexpression elevated CMTM2 expression in BGC823 cells (Figure 5F). Taken together, our results indicated that LINC01391 negatively modulated miR-12116 expression and positively modulated CMTM2 expression, and meanwhile miR-12116 negatively modulated CMTM2 expression.

### MiR-12116 and CMTM2 participated in the inhibitory effect of LINC01391 on cell invasion, migration and aerobic glycolysis in GC cells

Rescue assays were carried out via downmodulating miR-12116 or upmodulating CMTM2 expression in SGC7901 cells with LINC01391 knockdown. Transwell assays with or without Matrigel demonstrated that the cell invasion and migration were significantly accelerated via LINC01391 knockdown, and then it was partially reversed after transfection with miR-12116 inhibitor or p-CMTM2 (Figure 3A-3D). Meanwhile, the glucose uptake and lactate production were significantly accelerated via LINC01391 knockdown, and then it was partially reversed after transfection with miR-12116 inhibitor or p-CMTM2 (Figure 4A and B). Consistently, the GLUT1 and LDH-A expressions were significantly accelerated via LINC01391 knockdown, and then it was partially reversed after transfection with miR-12116 inhibitor or p-CMTM2 (Figure 5A, B and E). Thus, the above results demonstrated that miR-12116 inhibition or CMTM2 overexpression could partially reverse the promotive effects of LINC01391 knockdown on cell invasion, migration and glycolysis in SGC7901 cells.

Rescue assays were also carried out via upmodulating miR-12116 or downmodulating CMTM2 expression in BGC823 cells with LINC01391 overexpression. The cell invasion and migration were significantly hampered via LINC01391 overexpression, and then it was partially reversed after transfection with miR-12116 mimic or si-CMTM2 (Figure 3E-H). Meanwhile, the glucose uptake and lactate production were significantly hampered via LINC01391 overexpression, and then it was partially reversed after transfection with miR-12116 mimic or si-CMTM2 (Figure 4C and D). Consistently, the GLUT1 and LDH-A expressions were significantly hampered via LINC01391 overexpression, and then it was partially reversed after transfection with miR-12116 mimic or si-CMTM2 (Figure 5C, D and F). Thus, the above results demonstrated that miR-12116 upmodulation or CMTM2 knockdown could partially reverse the inhibitory effects of LINC01391 overexpression on cell invasion, migration and glycolysis in BGC823 cells. Overall, the above data suggested LINC01391 might modulate the cell invasion, migration and glycolysis of GC cells via miR-12116/CMTM2 axis.

## Discussion

In the current study, we demonstrated that LINC01391 expression was clearly decreased in GC tissues and cell lines. Mechanistically, we found that LINC01391 functions through a ceRNA-involved mechanism via competing with miR-12116, thus triggering CMTM2 expression and restraining the cell viability, invasion and aerobic glycolysis of GC cells. Our findings in this study revealed that LINC01391/miR-12116/CMTM2 axis is involved in GC progression, providing novel diagnostic, prognostic and therapeutic biomarkers and targets for GC.

The role of lncRNAs in GC formation and progression has gradually emerged 4,5. LINC01391 has recently been reported to exert an anti-oncogenic role in HCC cells 8. However, the role and mechanism of LINC01391 in GC progression has not been elucidated. In the current study, we here found that LINC01391 expression in GC tissues and cell lines was significantly decreased (Figure 1A and 1C), and low LINC01391 expression closely associated with lymph node metastasis, advanced TNM stage, invasion (Table 1) and poor overall survival ratio (Figure 1B). These results suggested that LINC01391 may exert an anti-oncogenic role in GC. However, the function involvement of LINC01391 in GC progression and its underlying molecular mechanisms still need to be further enhanced.

LncRNAs function as oncogenes or tumor suppressor genes via modulating cell proliferation, invasion and migration in various tumor cells 9,10. Here, we performed gain-of-function and loss-of-function experiments to study the effects of LINC01391 on cell biological behaviors of GC cells. The current results demonstrated that LINC01391 knockdown enabled GC cells to have higher capability for cell proliferation, higher tendency to invasion and migration *in vitro*, and accelerated tumor growth *in vivo* (Figure 2 and 3). These data revealed LINC01391 exerts the tumor-repressive role on cell biological behaviors of GC cells.

Previous studies demonstrated that aerobic glycolysis is implicated in the malignant behaviors of GC 11-13. Moreover, increasing data have revealed that lncRNAs can affect tumor formation and progression via modulating aerobic glycolysis 14. Here, we found that LINC01391 knockdown not only elevated the cell migration and invasion of GC cells (Figure 3), but also promoted glucose uptake and lactate production (Figure 4). Therefore, we speculated that LINC01391 may affect GC progression via modulating aerobic glycolysis. A number of enzymes have been confirmed to be involved in the aerobic glycolysis process, such as GLUT1, LDH-A 15,16. Furthermore, LDH-A can accelerate cell invasion, proliferation and glucose uptake of pituitary adenoma (PA) cells via upmodulating GLUT1 17. Thus, we here determined the GLUT1 and LDH-A expressions following modulation of LINC01391 expression, and found that LINC01391 knockdown accelerated GLUT1 and LDH-A expressions, and meanwhile LINC01391 overexpression restrained GLUT1 and LDH-A expressions (Figure 5), suggesting that LINC01391 may repress aerobic glycolysis of GC via upmodulating GLUT1 and LDH-A.

Accumulating evidence demonstrated that lncRNAs function as tumor promoters or tumor suppressors in various tumors, serving as ceRNAs via sponging miRNA to modulate downstream target gene expression 18-20. Based on the above research results, we supposed that LINC01391 may exert a tumor-repressive role in GC via functioning as ceRNAs for sponging downstream miRNAs. Using informatics analysis tools and qRT-PCR, we screened a few miRNAs sharing binding sites with LINC01391, and one of highest-ranked predicted potential target of LINC01391 was miR-12116 (Table 3, Figure 6A). Further, using RNA pull-down and luciferase activity assays, the binding relationship between LINC01391 and miR-12116 was identified in GC cells (Figure 7). Therefore, we speculated that LINC01391 might serve as a ceRNA to absorb miR-12116, and miR-12116 was chosen for our further experiments.

miRNAs function as oncogenes or tumor suppressor genes in GC progression via modulating their downstream target genes 21,22. However, so far, there is still no research report about miR-12116 in tumors. Herein we observed an obvious increase of miR-12116 expression in GC tissues (Figure 6B), and found that miR-12116 expression was negatively correlated with that of LINC01391 in GC tissues (Figure 6D). Furthermore, rescue experiments showed that upmodulated miR-12116 partially reversed the inhibitory effects of LINC01391 overexpression on the cell invasion, migration and aerobic glycolysis of GC cells, and meanwhile downmodulated miR-12116 partially reversed the promotive effects of LINC01391 knockdown on the cell invasion, migration and aerobic glycolysis of GC cells (Figures 3-5). These data suggested LINC01391 may restrain the progression of GC via targeting miR-12116. Further, we investigated how miR-12116 modulates the downstream target genes to further explore the role and molecular mechanism of miR-12116 in GC progression.

Since the roles and functions of miRNAs are realized via modulating their downstream target gene expression 23, we here conducted the bioinformatics analysis (Table 4) and verification experiments, and demonstrated that CMTM2 was the potential downstream target of miR-12116 (Figure 7). As a transcription factor, CMTM2 expression was downmodulated in HCC tissues, and low CMTM2 expression was associated with the poor prognosis of HCC patients, suggesting a potential tumor suppressor role of CMTM2 in HCC progression 24. Moreover, a significant novel mutation at CMTM2 was correlated with lymph node metastasis in diffuse-type gastric cancer (DGC), and CMTM2 overexpression significantly restrained cell proliferation in GC cells 25. Based on these studies and our above results, we hypothesized that LINC01391 may be partially required for CMTM2 to exert its anti-oncogenic effect in GC. To address this point, the current rescue experiments demonstrated that CMTM2 knockdown blocked LINC01391 overexpression-induced suppression of cell migration, invasion and glycolysis, while CMTM2 overexpression blocked LINC01391 knockdown-induced enhancement of cell migration, invasion and glycolysis (Figures 3-5). Thus, the current results suggested that LINC01391 may restrain cell migration, invasion and glycolysis of GC cells via modulating CMTM2.

Recently, increasing studies demonstrate that lncRNAs can directly interacts with miRNAs to modulate their downstream target expressions in GC progression 26,27. For instance, lncRNA MALAT1 directly interacts with miR-22-3p to modulate zinc finger protein 91 (ZFP91) expression in GC cells 26, and lncRNA ILF3-AS1 functions as a ceRNA via sponging miR-29a to elevate PTBP3 expression in GC cells 27. Interestingly, lncRNA LINC00504 acts as a molecular sponge for miR-1244 to modulate aerobic glycolysis in ovarian cancer 28. In the current study, bioinformatics analysis indicated potential binding sites in LINC01391 and miR-12116, as well as miR-12116 and CMTM2 3′-UTR (Figure 7), suggesting the possibility that LINC01391 may function as a ceRNA via interacting miR-12116 to elevate CMTM2 expression in GC cells.

**Figure 8:**
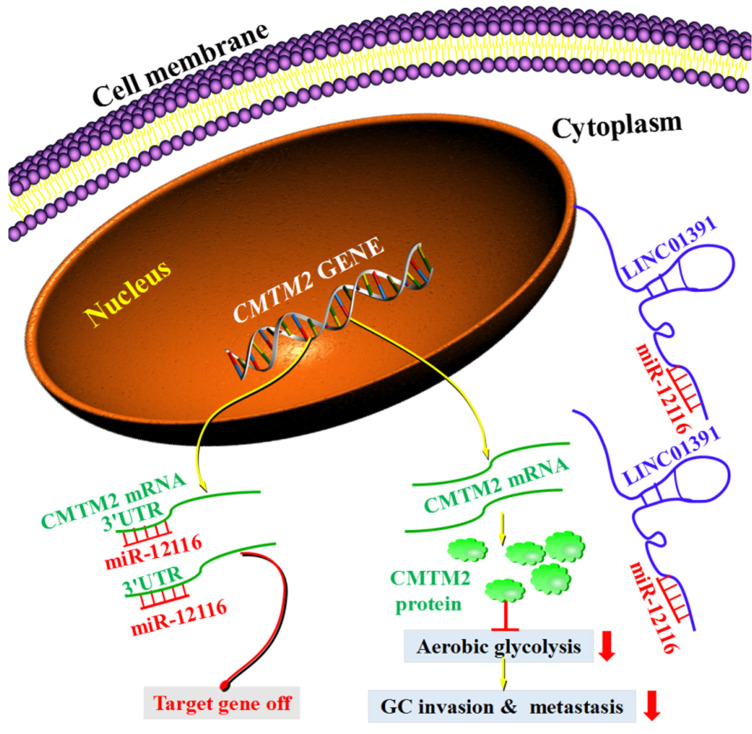
** Schematic model demonstrates the results of the study.** LINC01391 functions as a sponge of miR-12116 by adsorbing miR-12116 in the cytoplasm, and then miR-12116 is prevented from binding to CMTM2 3'-UTR, which leads to increased expression of CMTM2. Due to the increased CMTM2 expression in GC cells, aerobic glycolysis is weakened, and invasion and metastasis are inhibited. However, when the specific adsorption of LINC01391 is lacking, miR-12116 binds to CMTM2 3'-UTR, which inhibits the transcription and translation of CMTM2, resulting in a decrease in CMTM2 expression.

Further, we conducted experiments to confirm our hypothesis. The current data demonstrated that, LINC01391 expression was found to be inversely correlated with miR-12116 in GC tissues, while positively correlated with CMTM2 (Figure 6), which further strengthens the above-mentioned notion. Importantly, our current mechanistic experiments via pull-down and luciferase reporter assay showed the binding sites between CMTM2 3′-UTR and miR-12116, as well as LINC01391 and miR-12116 (Figure 7). Further, similar to miR-12116 upmodulation, LINC01391 knockdown restrained the expression of CMTM2, one of downstream target genes of miR-12116, whereas LINC01391 overexpression restrained the expression of miR-12116, leading to elevated expression of CMTM2 (Figures 5 and 7). Therefore, the effects of LINC01391 on GC cell biological behaviors can be partly explained with a ceRNA mechanism, by which LINC01391 functions as a ceRNA via interacting miR-12116, which attenuates CMTM2 expression via directly targeting its 3ʹ-UTR (Figure 8).

In conclusion, our current study revealed LINC01391 may be characterized as a tumor suppressor that was low expressed in GC tissues and cell lines. Furthermore, we suggested an important role for LINC01391 in aerobic glycolysis and tumor progression of GC via competitively binding and repressing miR-12116 to facilitate CMTM2 expression in GC. These findings in the current study imply that LINC01391 can function as a prognostic predictor for GC patients, and LINC01391/miR-12116/CMTM2 axis may be a potential therapeutic target for treating GC.

## Figures and Tables

**Table 1:** Correlations between LINC01391 expression and the clinical characteristics in GC patients

Characteristics	Case	LINC01391 expression		χ^2^	*P*-value
		Low	High		
**Gender**				0.404	0.525
Male	22	12	10		
Female	18	8	10		
**Age (years)**				0.902	0.342
≤65	19	8	11		
>65	21	12	9		
**TNM Stage**				8.120	0.004*
I, II	19	5	14		
III	21	15	6		
**Lymph node metastasis**				7.033	0.008*
Negative	14	3	11		
Positive	26	17	9		
**Tumor size**				5.227	0.022*
≤5 cm	15	4	11		
>5 cm	25	16	9		
**HP infection**				0.102	0.749
Negative	17	8	9		
Positive	23	12	11		

Note: The χ^2^ test was used for comparison between groups. Abbreviations: TNM, Tumor-Node-Metastasis; HP, Helicobacter pylori; *P <0.05.

**Table 2:** qRT-PCR primer sequences

Name	Primer sequence
LINC01391	F: 5′-TGGCACCCGCTATGTCCA-3′
	R: 5′-GTAGCAGGGATTCTGTCTG-3′
miR-3924	F: 5′-GCCATATGTATATGTGACTGCTACT-3′
	R: 5′-CTCTACAGCTATATTGCCAGCC-3′
miR-12116	F: 5′-GCCTTTGGTTCTTCTTAG-3′
	R: 5′-GCTCTGGGTTCTTCTTAG-3′
miR-939	F: 5′- CCTGGGGAGCTGAGGCT-3′
	R: 5′-CGCGGTCAGACACTGGG-3′
miR-2113	F: 5′-TGTGTGACAGGTACAGGGACA -3′
	R: 5′-TCAAAGTGGCATGTGACAGAG-3′
CMTM2	F: 5′-GCACCCCATCTTGAGGCTTAT-3′
	R: 5′-AGGTCAGAAATGGGCCACAG-3′
GAPDH	F: 5′-GCACCGTCAAGGCTGAGAAC-3′
	R: 5′-GCCTTCTCCATGGTGGTGAA-3′
U6	F: 5′-GCTTCGGCAGCACATATACTAAAAT-3′
	R: 5′-CGCTTCACGAATTTGCGTGTCAT-3′

**Table 3:** The predicted targets of LINC01391

Target Rank	Target Score	miRNA Name
1	90	miR-3924
2	89	miR-12116
3	85	miR-939
4	84	miR-2113
5	84	miR-138-5p
6	82	miR-4703-5p
7	82	miR-3942-5p
8	80	miR-6783-3p
9	80	miR-6890-3p
10	80	miR-2392

**Table 4:** The predicted targets for miR-12116

Target Rank	Target Score	Gene Symbol	Gene Description
1	98	KIAA1143	KIAA1143
2	97	ZDHHC15	zinc finger DHHC-type containing 15
3	95	VIRMA	vir like m6A methyltransferase associated
4	94	CETN3	centrin 3
5	94	CMTM2	CKLF like MARVEL transmembrane domain containing 2
6	92	ZNF148	zinc finger protein 148
7	92	ATP11C	ATPase phospholipid transporting 11C
8	92	EIF2AK3	eukaryotic translation initiation factor 2 alpha kinase 3
9	92	PRKD1	protein kinase D1
10	92	ZBTB41	zinc finger and BTB domain containing 41
